# Familial aggregation of subcutaneous panniculitis-like T cell lymphoma: A case report of monozygotic twin brothers

**DOI:** 10.1097/MD.0000000000022629

**Published:** 2020-10-16

**Authors:** Xun-Ze Shen, Shan-Lu Yu, Fang Liu, Zhou-Ye Luo

**Affiliations:** a PET/CT Center; b Department of Pathology, Shaoxing People's Hospital, the First Affiliated Hospital of Shaoxing University, Shaoxing, Zhejiang Province, China.

**Keywords:** familial inheritance, fluorine-18 fluorodeoxyglucose positron emission tomography/computed tomography, monozygotic twins, subcutaneous panniculitis-like T-cell lymphoma

## Abstract

**Rationale:**

Subcutaneous panniculitis-like T-cell lymphoma (SPTCL) is a rare subtype of cutaneous lymphoma, which was first defined as a clinical entity in 1991 as a cytotoxic T-cell lymphoma preferentially infiltrating subcutaneous tissue. Herein, we report 2 patients of SPTCL who are a pair of twin brothers.

**Patient concerns:**

The disease afflicted the monozygotic twin brothers at different time with an interval period of 5 years. The older twin brother had disease onset at 27 years of age. In June 2012, he developed prolonged fever accompanied by subcutaneous nodules in the left upper arm and left chest due to unknown origin. The younger twin brother had disease onset at 32 years of age. In June 2017, the younger brother presented with repeated high fever for more than 10 days, accompanied by head distension.

**Diagnosis:**

On August 7, 2012, skin biopsy was performed on the lesion of left upper arm of the older twin brother, and then, a diagnosis of subcutaneous panniculitis-like T cell lymphoma (SPTCL) was made. On June 19, 2017, the younger twin brother underwent whole-body fluorine-18 fluorodeoxyglucose positron emission tomography/computed tomography for diagnosis. Soon afterwards, abdominal subcutaneous nodule resection and biopsy was performed on June 28, 2018, and the specimen was diagnosed as SPTCL.

**Interventions:**

For the older brother, a total of 14 systemic chemotherapy sessions were performed from August 16, 2012, to September 21, 2014. For the younger brother, a total of 9 systemic chemotherapy sessions were performed from July 14, 2017, to March 8, 2018, then he was switched to oral chemotherapy with chidamide twice a week for 6 months.

**Outcomes:**

The older twin brother died in March 2015, the younger brother has recovered well and is no longer receiving any treatment

**Lessons:**

To the best of our knowledge, twin brothers both having this disease has never been previously reported. Moreover, some of the involved areas are also extremely rare detected by fluorine-18 fluorodeoxyglucose positron emission tomography/computed tomography at initial stage. It is beneficial to people to gain some new understanding for SPTCL by this special case and some extremely unusual involved sites in the younger twin brother.

## 1 Introduction

Subcutaneous panniculitis-like T-cell lymphoma (SPTCL) is a rare subtype of cutaneous lymphoma and belongs to a new subset of peripheral TCL, as determined by the World Health Organization. The SPTCL is characterized by infiltration of neoplastic cytotoxic T cells into subcutaneous tissue.^[1,2]^ It is a rare lymphoma that may affect younger patient and has an association with autoimmune disease. Patients usually present with multiple subcutaneous nodules and plaques involving the extremities or trunk.^[1,3–5]^ Dissemination to lymph nodes and other organs on initial presentation is very unusual.^[2,4,6]^

The SPTCL lesions can be assessed using various imaging modalities, such as ultrasonography, CT, magnetic resonance imaging, but positron emission tomography/computed tomography (PET/CT) detected many lesions with greater sensitivity than other image devices.^[4–9]^ PET/CT may therefore be useful in detecting the occult extracutaneous involvement when staging SCPTL as well as being a useful tool to quantify disease burden and response to treatment.^[5–8]^

Genetic, infectious, and environmental factors have been implicated as pathogenic causes for many types of lymphoma, and an inherited genetic lesion of the family could exist and predispose its affected members to the development of the SPTCL.^[10–13]^

## 2 Case presentation

### 2.1 Case 1

The older twin brother had disease onset at 27 years of age. In June 2012, the patient developed prolonged fever accompanied by subcutaneous nodules in the left upper arm and left chest due to unknown origin, and visited the rheumatology department of another hospital for treatment. The initial diagnosis was considered to be panniculitis and the patient was treated with methylprednisolone, but the therapeutic effect was poor. On August 7, 2012, skin biopsy was performed on the lesion of left upper arm, and the biopsy specimen showed infiltration of subcutaneous fatty tissue by various-sized atypical lymphocytes with partial apoptosis. Immunophenotype study revealed positive staining for CD3, CD5, CD8, and Granzyme-B, but negative staining for CD20, CD30, CD56, and CD79a. Hence, a diagnosis of subcutaneous panniculitis-like T cell lymphoma (SPTCL) was made.

**Figure 1:**
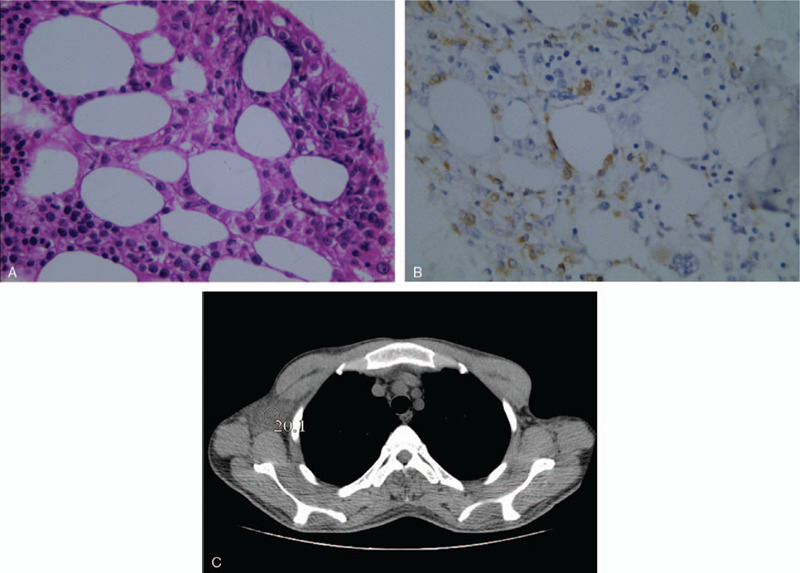
A, Photomicrograph of a bone marrow biopsy specimen showed various-sized neoplastic T cells with irregular and hyperchromatic nuclei around adipocytes (H&E, × 400). B, the immunohistochemical staining for CD3 was positive in the bone marrow biopsy (×400). C, Chest CT plain scan showed a diffuse soft tissue lesion involving subcutaneous fat tissue in the right axilla, with mean CT value of approximately 20HU.

A total of 14 systemic chemotherapy sessions were performed between August 16, 2012, and September 21, 2014. The patient first underwent 2 cycles of VDCLP (cyclophosphamide, vincristine, adriamycin, L-asparaginase, etoposide, methylprednisolone) regimen chemotherapy, but the effect was poor, with no significant improvement in body temperature. Between October 2012 and May 2013, the chemotherapy was changed to ESHAP (etoposide, cisplatin, Adriamycin, methylprednisolone) regimen, which lasted for 6 cycles. Three months later, new subcutaneous nodules appeared in the neck and left upper arm of the patient, and a biopsy indicated recurrence of lymphoma. In September 2013, the patient was given 1 cycle of GDP chemotherapy. In October 2013, another GDP chemotherapy cycle was given. Subsequently, the patient's body temperature improved and skin nodules were significantly reduced. In April 2014, the patient revisited our hospital due to fever and new systemic subcutaneous nodules. The bone marrow biopsy on the left posterior superior iliac spine was performed, which demonstrated numerous atypical lymphocytes, and a diagnosis of T-cell lymphoma involving the bone marrow was considered (Fig. 1A). The infiltrating cells had a CD3+ (Fig. 1B), CD5-, CD20-, CD31-, CD34-, CD45+, CD68+, CD79a-, CK-, and MPO+ phenotype. Chest CT plain scan on April 10, 2014, showed a diffuse soft tissue lesion involving subcutaneous fat tissue in the right axilla, with mean CT value of approximately 20HU (Fig. 1C). Considering the recurrence of lymphoma, the patient was given 1 cycle of MA (mitoxantrone, Adriamycin) chemotherapy, 1 cycle of MAP (mitoxantrone, Adriamycin, dexamethasone) chemotherapy in July 2014, and 1 cycle of MAEP (mitoxantrone, Adriamycin, etoposide, dexamethasone) regimen in August and September 2014, respectively. The patient died in March 2015.

### 2.2 Case 2

The younger twin brother had disease onset at 32 years of age. In June 2017, the patient was referred to our hospital for treatment due to repeated high fever for more than 10 days, accompanied by head distension. Blood test showed WBC count 3.13 × 10^9^/L, neutrophil percentage 67.4%, CRP < 0.50 mg/L, and CEA 6.06 ng/mL. 555

On June 19, 2017, the patient underwent whole-body fluorine-18 fluorodeoxyglucose positron emission tomography/computed tomography for diagnosis, with calculation of the maximum standardized FDG accumulation in pathological foci (standard uptake value, SUVmax). The PET/CT showed multiple systemic metabolically active lesions (in subcutaneous fat of the right occiput, the left armpit, the right lower abdomen and both buttocks, in inter-muscular fat of the left superior chest wall, in bilateral extra-pleural and extra-peritoneal fat, in right intra-renal dispose capsule, and in the mesentery of the colorectum), with increased fat density and FDG uptake demonstrated on whole body maximum intensity projection and axial images (Fig. 2 A–F). PET/CT also showed a slightly enlarged lymph node with increased FDG uptake in the right axilla (Fig. 2 G). The SUVmax of these lesions varied from 1.9 to 8.4.

**Figure 2:**
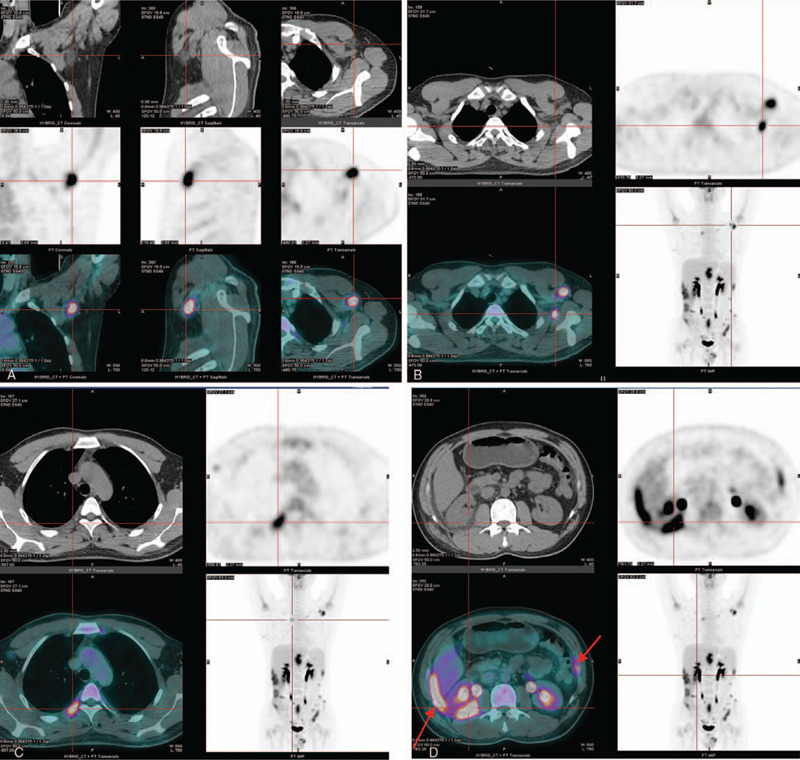
A, The images of fluorine-18 fluorodeoxyglucose positron emission tomography/computed tomography showed a hyper-density plaque with remarkably increased uptake of FDG in the left axilla (SUVmax=7.232). B, a hyper-density FDG-avid lesion within the fat space among the muscles of the left upper chest wall was found. C, the images demonstrated lesion in the right extra-pleural fat with increased density and FDG uptake (SUVmax= 4.506). D, the images showed multiple lesions in the fatty capsule of right kidney and bilateral extra-peritoneal fat, with increased density and FDG uptake. E, the images showed infiltrative lesions within subcutaneous fat tissue in the right hip and colon mesentery (indicated by the arrows). F, FDG-PET images showed hypermetabolism in the areas of mesorectum with slight increase in density. G, a slightly enlarged lymph node presented with abnormal F-18 FDG uptake in the right axilla (SUVmax= 1.944, 9 × 7 mm).

**Figure 2 (Continued):**
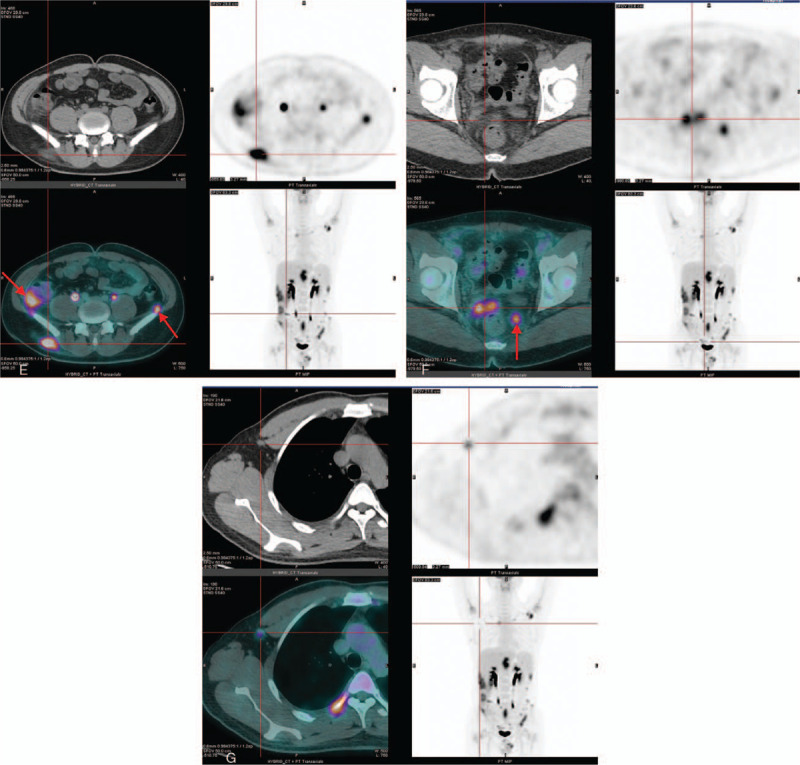
A, The images of fluorine-18 fluorodeoxyglucose positron emission tomography/computed tomography showed a hyper-density plaque with remarkably increased uptake of FDG in the left axilla (SUVmax=7.232). B, a hyper-density FDG-avid lesion within the fat space among the muscles of the left upper chest wall was found. C, the images demonstrated lesion in the right extra-pleural fat with increased density and FDG uptake (SUVmax= 4.506). D, the images showed multiple lesions in the fatty capsule of right kidney and bilateral extra-peritoneal fat, with increased density and FDG uptake. E, the images showed infiltrative lesions within subcutaneous fat tissue in the right hip and colon mesentery (indicated by the arrows). F, FDG-PET images showed hypermetabolism in the areas of mesorectum with slight increase in density. G, a slightly enlarged lymph node presented with abnormal F-18 FDG uptake in the right axilla (SUVmax= 1.944, 9 × 7 mm).

Abdominal subcutaneous nodule resection and biopsy was performed on June 28, 2018, at another hospital, and the specimen was diagnosed as SPTCL. The tumor cells expressed CD3, CD5, CD7, CD8, granzyme B, TIA-1, and ki-67 63%, but not CD20, CD4, CD138, or CD56.

A total of 9 systemic chemotherapy sessions were performed between July 14, 2017, and March 8, 2018. The patient underwent 2 cycles of pegaspargase with CDOP (vindesine sulfate, cyclophosphamide, liposome of doxorubicin hydrochloride, prednisone) chemotherapy, followed by 2 cycles of pegaspargase with CHOPE (etoposide, vindesine sulfate, cyclophosphamide, liposome of doxorubicin hydrochloride, dexamethasone sodium phosphate) chemotherapy. Based on the patient's abdominal CT findings, lactate dehydrogenase level, recent fever, and clinical progression, the chemotherapy regimen was changed to 2 cycles of GDP (cisplatin, dexamethasone sodium phosphate, gemcitabine) regimen on October 23, 2017. On January 2, 2018, systemic PET/CT indicated tumor progression, and the chemotherapy regimen of the patient was subsequently adjusted to 3 cycles of cladoxabine + mitoxantrone + dexamethasone. Then the patient was switched to oral chemotherapy with chidamide twice a week for 6 months. No active lesion of lymphoma was found in PET/CT reexamination at December 2018 during the treatment of chidamide. The patient has recovered well and is no longer receiving any treatment.

Ethical approval was not required, because this paper is a case report with the clinical information of the patient. Informed written consent of publishing this case report and accompanying images were obtained from the second patient who was on behalf of himself and his late twin brother.

## 3 Discussion

Subcutaneous panniculitis-like T-cell lymphoma, as strictly defined by World Health Organization-European Organization for Research and Treatment of Cancer classification, is a rare cytotoxic α/β T-cell lymphoma (<1% non-Hodgkin lymphoma), characterized by primary involvement of subcutaneous tissue mimicking panniculitis.^[1,2]^

Histologically, SPTCL is characterized by a lymphohistiocytic infiltrate, primarily confined to the interstitium of fat lobules in a pattern resembling lobular panniculitis. Lymphoid cells tend to surround individual adipocytes and have a characteristically rimmed appearance.^[3]^ The typical case shows positive staining for CD3, CD8, TIA-1, granzyme, and BetaF1 and negative staining for CD4, TCR gamma, CD56, or EBV by in situ hybridization.

SPTCL occurs in adults as well as in young children, and it has a slight female predilection. The median age at diagnosis is 36 (range 9–79) years, with approximately one-fifth of cases occurring in patients younger than 20 years.^[1,3]^ SPTCL may be associated with autoimmune disease. Patients usually present with solitary or multiple subcutaneous nodules and plaques involving the extremities or trunk, with constitutional symptoms that may include fever, myalgia, chills, and weight loss. Most patients show several abnormalities, primarily including anemia, leukopenia, and/or thrombocytopenia, as well as increased levels of liver enzymes. The disease may be complicated by a hemophagocytic syndrome, which is generally associated with a rapidly progressive course.^[4,5]^

Dissemination to lymph nodes and extra-cutaneous sites on initial presentation is very rare.^[2,4,6]^ Babb A reported a case of SPTCL with lesions involving the fat space between the muscles of bilateral chest wall. In our second case, the fat space in the muscles of upper left chest wall was involved and bilateral extra-pleural fat lesions were also observed, which has not been previously reported. Involvement of intra-abdominal fat in SPTCL has been described in recent years.^[2,4,7,8]^ However, these studies only reported the involvement of mesenteric fat. In our second case, not only colorectal mesentery had several lesions but also the patient's right renal dispose capsule was diffusely involved, and the bilateral extra-peritoneal spaces were also involved, which has not been previously reported. Lymph node involvement is also rare. Kim JW reported 8 patients of SPTCL, of which only 1 showed involvement of the iliac lymph nodes. Lack of lymph node involvement may obscure the diagnosis of lymphoma on CT. Lymph node lesion was also seen in our case 2, which was located in the right axilla.

SPTCL is characterized by multiple, nodular, or diffuse soft tissue lesions involving subcutaneous fat tissue. Subcutaneous fat necrosis is the primary radiological imaging feature of SPTCL.^[4,6]^ The imaging appearance of fat necrosis is mainly determined by the extent of the associated fibrotic reaction, along with the volume of liquefied fat and the presence of calcifications. When minimal fibrosis occurs, fat necrosis appears as a radiolucent mass or as an oil cyst, and the reparative fibrotic process may entirely replace the necrotic fat, resulting in a focal dense mass on plain X-ray or CT image.^[3,4,9]^

On FDG-PET, SPTCL lesions appear as multiple foci of FDG uptake, and often show dramatic resolution after chemotherapy.^[2,6–8]^ Our second case also showed FDG-avid lesions in the initial pre-treatment PET, which correlated with a high index of proliferative activity of lymphoid cells. The clinical application value of PET/CT in SPTCL diagnosis is as follows:

(1) FDG PET/CT can clearly show the extent and degree of SPTCL lesions, which provides valuable information for detecting occult lesions.
(2) FDG PET/CT can guide the selection of clinical biopsy sites and improve the accuracy of puncture.
(3) Chemotherapy is currently the main treatment for SPTCL. There are many chemotherapies for SPTCL, which offers a wide range of options for clinicians. It is necessary to monitor the curative effect of chemotherapy in the early stage and adjust the chemotherapy regimen in time. FDG PET/CT can play a very important role in disease staging and monitoring of treatment response, which help in choosing the first-line therapy.
(4) PET/CT can be used for follow-up and prognosis of SPTCL.

In this report, the 2 patients were monozygotic twin brothers. This is the first report of twins with SPTCL. Gau JP reported a familial occurrence of SPTCL, in which the disease afflicted a boy and his non-twin elder sister at different times with an interval period of 11 years, but their eldest brother did not have any relevant disease.^[10]^ Schneider BF reported a familial occurrence of cutaneous T cell lymphoma (CTCL) in 1995, in which the patients were monozygotic twin sisters.^[11]^ Although the CTCL in that report was not SPTCL, it suggests that T cell lymphoma may have familial inheritance. Genetic, infectious, and environmental factors have been implicated as pathogenic causes for CTCL.^[11]^

Familial aggregation of NHL has been reported in 3.5% of newly diagnosed patients, suggesting the existence of a hereditary subset.^[12]^ Highest lifetime risks were found when NHL was diagnosed in a twin (3%–13%), a sister (1.9%–2.5%) or 2 first-degree relatives (2.1%).^[13]^

In summary, SPTCL is characterized by multiple nodular or diffuse soft tissue lesions involving subcutaneous fat tissue, but it can also involve lymph nodes, and extra-cutaneous sites. The presence of multiple subcutaneous nodules detected by CT throughout the body may be an important finding to suggest a diagnosis of SPTCL. FDG PET/CT detected many lesions with greater sensitivity than did physical examination or CT in this report. FDG PET was valuable in monitoring the treatment response and detecting the extra-cutaneous lesion in SPTCL. SPTCL can have familial inheritance, and a family history of SPTCL diagnosed at any age can be considered as a risk factor for SPTCL in relatives, especially when SPTCL was diagnosed in a twin, a sister or 2 first-degree relatives.

## Author contributions

Shen XZ designed the research; Shen XZ and Luo ZY wrote the paper; Yu SL and Liu F provided and analyzed the pathological images.

**Conceptualization:** Xun-Ze Shen.

**Formal analysis:** Zhou-Ye Luo.

**Resources:** Shan-Lu Yu, Fang Liu.

**Writing – original draft:** Xun-Ze Shen.

**Writing – review & editing:** Xun-Ze Shen.
